# COVID-19 Lesson for Respiratory Syncytial Virus (RSV): Hygiene Works

**DOI:** 10.3390/children8121144

**Published:** 2021-12-06

**Authors:** Andrea Gastaldi, Daniele Donà, Elisa Barbieri, Carlo Giaquinto, Louis J. Bont, Eugenio Baraldi

**Affiliations:** 1 Division of Pediatric Infectious Diseases, Department of Women’s and Children’s Health, University of Padua, 35128 Padua, Italy; a.gastaldi.91@gmail.com (A.G.); elisa.barbieri@unipd.it (E.B.); carlo.giaquinto@unipd.it (C.G.); 2 Department of Pediatrics, Woman and Child Hospital, University of Verona, 37126 Verona, Italy; 3 Department of Pediatrics, Wilhelmina Children’s Hospital, University Medical Center Utrecht, 3584 CX Utrecht, The Netherlands; lbont@umcutrecht.nl; 4 Respiratory Syncytial Virus Network (ReSViNET) Foundation, 3703 CD Zeist, The Netherlands; 5 Neonatal Intensive Care Unit, Department of Women’s and Children’s Health, University of Padua, 35128 Padua, Italy; eugenio.baraldi@unipd.it; 6 Fondazione Istituto di Ricerca Pediatrica, 35127 Padua, Italy

**Keywords:** respiratory syncytial virus, COVID-19, lower respiratory tract infection, children, infant primary prevention, hygiene

## Abstract

Respiratory syncytial virus (RSV) is a leading cause of acute lower respiratory tract infections (LRTIs) in infants worldwide. The global direct medical cost associated with RSV LRTIs reaches billions of dollars, with the highest burden in low–middle-income countries. Many efforts have been devoted to improving its prevention and management, including both non-pharmaceutical and pharmaceutical strategies, often with limited routine use in high-income countries due to high costs. During the ongoing COVID-19 pandemic, a dramatic decrease in RSV infections (up to 70–90%) has been reported around the globe, directly related to the implementation of containment measures (face masks, hand hygiene, and social distancing). Primary prevention has demonstrated the highest cost effectiveness ratio in reducing the burden of a respiratory infection such as RSV, never reached before. Thus, we emphasize the importance of non-pharmaceutical preventive hygiene measures that should be implemented and maintained even after the COVID-19 outbreak.

## 1. Introduction

Bronchiolitis is defined as an acute lower respiratory tract infection (LRTI) of the small airways, primarily affecting infants <12 months old. It is typically triggered by viral infections, with the respiratory syncytial virus (RSV) being the most common cause. With seasonal outbreaks worldwide, RSV has been demonstrated to be highly contagious and to be associated with a more severe clinical presentation, particularly in high-risk patients, such as preterm infants or children with comorbidities [1,2,3,4,5].

In 2005, it was estimated that 33.8 million new RSV-associated acute LRTIs occurred in children <5 years old (22% of total acute LRTI episodes), with around 3.4 million cases categorized as severe and thus necessitating hospital admission. In the same paper, mortality was reported in between 66,000 and 199,000 children, and 99% of deaths occurred in developing countries [6].

Ten years later, the burden of disease in children younger than five years of age did not vary significantly, with 3.2 million hospital admissions, with an overall RSV mortality of around 118,200. In 2015, 2.8 million RSV infections were estimated in children <5 years old in high-income countries, resulting in around 383,000 hospital admissions and 3300 deaths. In the same setting, the RSV hospitalization rate was 26.3, 11.3, and 1.4/1000 persons in children less than five months, 6–11 months, and 12–59 months of age, respectively. Mortality rates were 0.2%, 0.9%, and 0.7%, respectively [7,8].

In a systematic review and meta-analysis, the global annual rate of RSV hospitalization among children <5 years old was 4.4 per 1000 children-years, with the highest rate among children <6 months old and premature infants <1 year old (20.0 and 63.9 per 1000, respectively) [9].

Severe RSV disease is associated with caregiver loss of work productivity, increased hospitalization costs due to intensive care unit admission and ventilatory support, and long-term complications, including abnormal lung function, with bronchial hyper-responsiveness and recurrent wheezing [10]. Many studies have identified a substantial economic burden on healthcare systems, governments, and society [11,12,13,14].

In France, Butel and colleagues calculated an average total cost per patient of around EUR 2000 of a first episode of acute bronchiolitis, mostly due to hospitalization costs [11].

In a recent systematic review and meta-analysis, the global direct medical cost associated with inpatient and outpatient RSV LRTIs in 2017 was estimated to be approximately EUR 4.82 billion, with EUR 3.13 billion in developing countries alone. In addition, direct non-medical costs and indirect costs would further add 8.7% and 31.6%, respectively, to the total direct medical costs [12].

RSV is biologically unique inasmuch as there is an increasing amount of evidence indicating that early-life RSV infection is associated with the onset of wheezing/asthma later on, with implications for long-term respiratory health and medical costs [15,16].

RSV prevention is essential to reduce the burden related to further sequelae [17,18,19].

Indeed, bronchiolitis is self-limiting in most cases. Several evidence-based guidelines exist assessing supportive care as the key clinical management strategy, primarily directed to monitoring disease progression, assisting feeding with fluid administration, and respiratory support. Specific pharmaceutical interventions are not routinely recommended because they lack proven benefits and increase care costs and potential adverse effects [2,4,5,20,21,22,23].

Nonetheless, wide variations in diagnostic test utilization and unnecessary non-evidence-based treatments are still consistent in primary care and hospital settings, which are not in line with guidelines [24,25,26].

In a retrospective cohort study in 38 pediatric emergency departments (EDs), more than 30% of infants hospitalized with bronchiolitis received non-evidence-based treatments, including administration of inhaled epinephrine, salbutamol, hypertonic saline, or systemic corticosteroids [27].

While inhaled β2-agonist and glucocorticoid prescriptions seem to have been reduced over time, antibiotics are still widely prescribed with a potential increase in antimicrobial resistance, prolonged hospitalization, and further preventable costs [28,29].

Many efforts have been devoted to improving prevention and management strategies that could reduce costs and pressure on healthcare systems in high-income and developing countries, but results have been scarce.

The COVID-19 pandemic has been demonstrating the importance of primary prevention against communicable diseases. The first clear evidence of clinical benefit from hand hygiene came from Ignaz Semmelweis already in 1840. Nevertheless, the link between handwashing and the spread of disease was established only two decades ago. Since then, hand hygiene has been the cornerstone of all infection prevention and control programs, including diarrhea. Washing hands with soap can reduce the risk of diarrheal diseases by 42–47%, and interventions to promote handwashing might save millions of lives [30,31]. Hand hygiene is a simple and cost-effective health intervention. According to Chen and colleagues, every EUR 1 spent on hand hygiene promotion will result in a EUR 23.7 benefit [32].

## 2. Effects of Non-Pharmaceutical Interventions on Bronchiolitis Management

Implementation and adherence to international guidelines that discourage non-evidence-based use of pharmacotherapy and diagnostic testing (often due to the clinician’s uncertainties around the diagnosis rather than clinical evidence) are essential to reduce unnecessary costs and resource utilization and improve the value of care provided to infants with bronchiolitis.

A segmented time-series analysis, examining ED visits of 2929 patients with bronchiolitis, estimated a total cost saving of EUR 196,409 (mean cost per patient reduced by EUR 197) over two bronchiolitis seasons after guideline implementation in the US, with an absolute reduction of 23% in chest X-ray and 7% in albuterol use [33].

Many efforts have been devoted to improving and standardizing bronchiolitis care, from primary care to the pediatric ward and pediatric intensive care unit (PICU) settings.

A recent multicenter cluster-randomized clinical trial set in Australia and New Zealand demonstrated the effectiveness of targeted interventions over passive dissemination of an evidence-based bronchiolitis guideline in improving the treatment of infants with bronchiolitis. The interventions included site-based clinical leads, stakeholder meetings, a train-the-trainer workshop, targeted educational delivery, other educational and promotional materials, audit, and feedback [34].

An evidence-based management protocol and interactive sessions have also been implemented in Spain to reduce unnecessary medications in infants with bronchiolitis in primary care. These included online interactive educational meetings for clinicians to revise epidemiological data, diagnostic criteria, and international recommendations and to share data [35].

Despite the absence of documented bacterial coinfections, children with bronchiolitis often receive inappropriate antimicrobial therapies, increasing costs and the risk of antimicrobial resistance [36]. Different antibiotic stewardship interventions have been developed to avoid antibiotic overuse. Most protocols are based on biomarkers, such as procalcitonin and C-reactive protein, to determine the probability of bacterial coinfections [37,38]. Integrating these biomarkers in a clinical pathway led to a significant reduction in antibiotic prescriptions in patients hospitalized for bronchiolitis in Italy [39].

Thus, evidence-based protocols supported by educational activities are associated with significant changes in physicians’ prescribing habits and can improve children’s outcomes.

## 3. Effects of Pharmaceutical Interventions on RSV Prophylaxis

To date, no target therapies have been discovered for treatment. Thus, the most important measure to limit its spread and risk of severe disease is prevention.

In the past decade, multiple vaccines and other therapeutic agents have been developed in active clinical trials. However, palivizumab still represents the only intervention licensed for RSV prevention in high-risk infants since 1998 [8,40].

Palivizumab is a recombinant humanized monoclonal antibody that can be administrated intramuscularly to prevent serious LRTIs caused by RSV. It acts by binding the RSV envelope fusion protein (RSV F) on the virus’s surface and blocking a critical step in the membrane fusion process, providing passive immunity against RSV. Therefore, several guidelines recommend palivizumab for the prophylaxis of RSV infection in preterm infants, infants with congenital heart disease, and other high-risk populations [4,8,41]. Palivizumab still represents a valuable prevention strategy for RSV in complex chronic conditions, as numerous cost effectiveness analyses pointed out. Indeed, recent systematic reviews reported incremental cost effectiveness ratios ranging from USD 15,000 to 140,000/quality-adjusted life-years (QALY) for children with congenital heart disease, USD 31,000 to 38,000/QALY for those with chronic lung disease, and USD 800 to 800,000/QALY for preterm infants (≤35 gestational weeks) [42].

A new anti-F protein monoclonal antibody, nirsevimab, provides protection throughout the whole RSV season, with a single dose administered at the beginning of the epidemic season. A recent study recruiting 1500 preterm infants treated with nirsevimab resulted in fewer medically attended RSV-associated lower respiratory tract infections and hospitalizations than placebo in healthy preterm infants [43].

Several RSV vaccine candidates that target infants and pregnant women in the third trimester are in clinical development. On the horizon, RSV immunization represents a promising strategy for the prevention of RSV infections [44,45,46].

## 4. Impact of COVID-19 Containment Measures on Bronchiolitis

RSV, as with other viruses, is transmitted through air droplets and aerosols and by direct or indirect contact with contaminated objects or surfaces.

Following the start of the COVID-19 pandemic, many containment measures have been adopted to reduce the spread of the virus, such as physical distancing and the compulsory use of face masks and hand hygiene. These measures have helped contain the transmission of SARS-CoV-2 and inevitably influenced the spread of other viruses, reducing the burden of other acute respiratory infections (ARIs). Data registered a lower incidence of ARIs both in children and adults during the implementation of COVID-19 containment measures, also including influenza and asthma exacerbations, which are commonly triggered by viral infections and represent a frequent reason for hospital admission [47,48,49,50].

**Figure 1:**
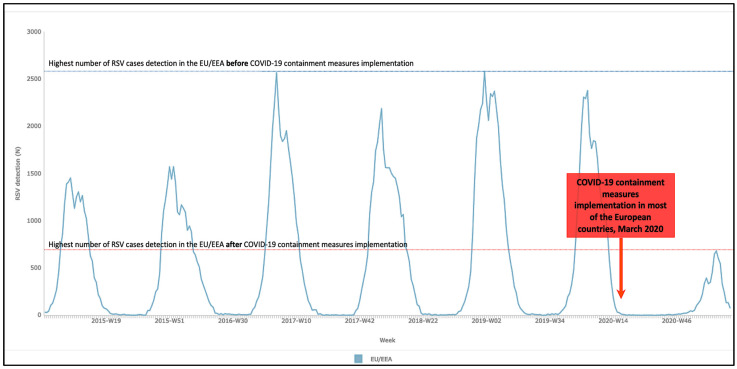
Effects of implementation of containment measures on RSV incidence (adapted from https://atlas.ecdc.europa.eu/public/index.aspx, accessed on 31 July 2021).

An exceptional decrease in bronchiolitis incidence has been reported from different countries (Italy, Belgium, France, Brazil), highlighting the unprecedented impact of prevention measures for SARS-COV-2 on RSV infections. The European Centre for Prevention and Diseases Control registered a fall in the detection of RSV from 2000–2500 cases per week at peak incidence during the previous four seasonal outbreaks (2016–2017, 2017–2018, 2018–2019, 2019–2020) to less than 700 cases per week in 2020–2021 (Figure 1) [51].

A retrospective analysis in a tertiary pediatric ED in Italy reported a decrease in the ED admission rates for acute bronchiolitis during the COVID-19 outbreak, regardless of changes in circulating respiratory viruses. The authors observed an average drop of 84% in the rate of acute bronchiolitis in 2020–2021 compared with bronchiolitis seasons of the previous five years [52].

Another third-level Italian pediatric hospital registered a drastic reduction in admissions due to bronchiolitis (95% decrease) in 2020–2021, without peak incidence, compared to the previous two seasonal outbreaks [53].

The bronchiolitis burden dramatically decreased during the second COVID-19 wave in France, with hospitalization being 82.5% lower than predicted values and a further 85.3% reduction in PICU admissions. The authors also highlighted that local physical distancing measures were applied in France, but children continued to attend daycare centers and schools. Therefore, their findings challenge the widely accepted notion that children are the main vectors of bronchiolitis viruses and suggest that adults may play an essential role in spreading the viruses [54,55].

In Belgium, researchers reported a >99% reduction in the number of registered RSV cases (only 20 in the whole country) before the expected end of the peak in 2020, compared to the previous three years. In the city of Antwerp, they counted 92.5% fewer bronchiolitis hospitalizations during the same period [56].

In Brazil, social distancing and non-pharmaceutical interventions significantly reduced hospitalization for ARIs in children with acute bronchiolitis by 70% up to 93% in most scenarios [49,57].

In South Africa, a facility-based surveillance revealed a decline in RSV and influenza virus detection with strengthening of the most stringent COVID-19 control strategies, compared with previous years. However, a subsequent increase in RSV was observed after the typical season, possibly reflecting the easing of measures [58].

Indeed, further cost analysis studies would document a proportional drop in related medical costs in terms of billions of euros/dollars in those countries registering a drastic reduction in RSV hospitalization (up to 70–90%) during the containment measures following COVID-19.

## 5. Primary Prevention

RSV infection is a worldwide issue in terms of mortality, morbidity, and healthcare costs. Therefore, the prevention of RSV infection has been declared a key priority by the World Health Organization (WHO) [59].

The introduction of palivizumab represented an important resource to protect the frailest patients with a higher risk of severe disease and complications from RSV, as the cost effectiveness studies have shown. However, while this constitutes a valuable prevention tool in high-income countries, it is less sustainable in developing countries with limited resources due to its high cost, its requirement for four to five injections per child per season, and the challenge of defining the exact timing and duration of the RSV season in subtropical and tropical regions [14,60].

Transmission of the virus predominantly occurs through inoculation of nasopharyngeal or ocular mucous membranes after direct contact with virus-containing secretions; thus, general measures that decrease inoculation (handwashing, cough hygiene, avoidance of smoke exposure, etc.) can limit its spread.

The COVID-19 pandemic has shown how primary prevention, limiting exposure to an infectious agent, has been the most powerful strategy to reduce the contagion and spread of SARS-CoV-2. Protocols have been developed for this purpose by adopting specific protective devices and environmental measures (for infected objects, equipment, or surfaces), especially in the workplace [61]. Indeed, pending other pharmacological tools (i.e., vaccination or target therapies), efforts against numerous other microbiological agents, including RSV, can benefit from these precautions.

According to the RSV transmission mode [62,63,64], massive hand hygiene campaigns were the most effective intervention in reducing the spread of infection. Further weaker evidence shows that even masks contributed to limiting the spread of droplets and reducing direct contact of hands with the nose and mouth.

Further epidemiological studies may show the actual incidence of RSV and possibly analyze the costs averted during the pandemic. Indeed, data available from many countries already highlight a drastic reduction in infection (with rates decreased by over 70%). However, a similar decrease has never been achieved, even after the introduction of palivizumab.

Primary prevention strategies are based on effective measures such as the systematic use of masks, hand and surface hygiene, and physical distancing, with an undoubtedly favorable cost/benefit ratio. ARIs become more easily preventable with only non-pharmaceutical initiatives.

Regardless of acute infections, the effects of RSV also involve long-term lung health. In a South African cohort study of 1143 infants from birth to 2 years of age, early-life RSV LRTIs were associated with recurrent LRTIs (three times higher than non-RSV), particularly recurrent wheezing [65]. Considering the drastic reduction in exposure to RSV during the pandemic for most children between 0 and 18 months, these strategies could positively reduce the risk of future recurrent wheezing illnesses.

The usability and the low cost make primary prevention strategies more accessible, limiting the burden of the disease and the risk of mortality, especially in resource-limited settings where access to palivizumab and secondary prevention strategies is restrained. A bottle of alcohol-based hand sanitizer costs only a few dollars, making hand hygiene an affordable and feasible health intervention worldwide, saving thousands of dollars in single hospital admissions.

## 6. Future Research Directions

A further lesson that can be learned from the COVID-19 pandemic experience is that more straightforward tools can produce positive effects at a lower cost. Primary prevention cannot be separated from a constant effort to implement health education worldwide regarding hand hygiene and face masks. It can also be related to a sizable reduction in RSV long-term sequelae, particularly recurrent LRTIs and wheezing. We should take advantage of this opportunity for the future as people are now used to handwashing. Hand hygiene of family members is essential to reduce the risk in the first few months of life, even if this practice could be challenging for the younger ones. Moreover, because aspiration of oral bacteria induces the expression of angiotensin-converting enzyme 2, a receptor for SARS-CoV-2, and production of inflammatory cytokines in the lower respiratory tract, poor oral hygiene can also lead to COVID-19 aggravation [66,67].

It becomes imperative to keep investing in primary prevention in order not to undo the benefits achieved during the pandemic against most respiratory pathogens, especially for most vulnerable children.

The risk of a potential rebound outbreak of respiratory infection following the easing of lockdown restrictions could cause new dramatic scenarios [68]. Indeed, some authors hypothesized greater RSV epidemics in the coming seasons due to the re-circulation of RSV within immunologically naïve populations of infants born from mothers who have not reinforced their immunity to the virus [69].

## 7. Conclusions

RSV is a significant cause of morbidity and mortality in infants worldwide, with the highest burden in low–middle-income countries. Palivizumab is fundamental for preventing serious RSV LRTIs in high-risk infants, but its high cost limits its routine use. The ongoing pandemic has demonstrated the higher cost effectiveness ratio of primary prevention (hand hygiene, face masks, etc.) in reducing the burden of RSV.

Thus, besides immunization strategies, we strongly emphasize implementing non-pharmaceutical preventive hygiene measures even after the COVID-19 pandemic to limit the spread of RSV worldwide.
